# The optimal dose of brisk walking for improving blood pressure in hypertensive patients: a systematic review and bayesian meta-analysis of randomized controlled trials

**DOI:** 10.7717/peerj.21478

**Published:** 2026-06-30

**Authors:** Jialin Wang, Li Ma, Qihan Lin, Yikun Yin

**Affiliations:** 1 School of Physical Education, Jining University, Jining, China; 2 Institute of China Sports Development, Beijing Sport University, Beijing, China; 3 Inner Mongolia University of Finance and Economics, Hohhot, China; 4 College of Physical Education and Health, Longyan University, Longyan, China; 5 School of Sport Human Science, Beijing Sport University, Beijing, China

**Keywords:** Brisk walking, Hypertension, Blood pressure, Dose-response, Bayesian meta-analysis

## Abstract

**Background:**

Hypertension is one of the most common chronic diseases worldwide. Brisk walking is regarded as a safe and effective exercise modality for blood pressure reduction; however, its optimal exercise dose remains unclear. This study aims to systematically evaluate the effects of brisk walking on blood pressure in patients with hypertension using a Bayesian meta-analysis, and to determine the dose-response relationship and the optimal exercise dose range.

**Methods:**

Randomized controlled trials (RCTs) were systematically searched in China National Knowledge Infrastructure (CNKI), VIP Information (VIP), Wanfang Data, CBM, PubMed, EBSCO (MEDLINE), Embase, the Cochrane Library, and Web of Science from database inception to December 30, 2025. Bayesian hierarchical random-effects models were constructed using the brms, metafor, and dosresmeta packages in R software (version 4.4.3) to perform pairwise comparisons and dose-response meta-analyses. The risk of bias was assessed using the Cochrane Risk of Bias 2 (RoB 2) tool.

**Results:**

A total of 17 RCTs involving 1,493 patients with hypertension were included. Brisk walking significantly reduced systolic blood pressure (Hedges’ g = − 0.48, 95% CrI [−0.54 to −0.43]) and diastolic blood pressure (Hedges’ g = − 0.51, 95% CrI [−0.58 to −0.44]), both representing moderate effect sizes. A “U” shaped dose-response relationship was observed for systolic blood pressure, with the optimal dose at approximately 460 MET (Metabolic Equivalent) ⋅ min/week (Hedges’ g = − 1.00, 95% CrI [−1.54 ∼−0.46]), indicating a large effect size. Diastolic blood pressure exhibited a negative dose–response relationship, with the most significant improvement also observed at approximately 620 MET min/week (Hedges ’ g = − 0.55, 95% CrI [−1.06 ∼−0.03]), corresponding to a substantial effect.

**Conclusion:**

Brisk walking significantly improves blood pressure in patients with hypertension and demonstrates a precise dose-response relationship. A brisk walking regimen of four sessions per week, ≤ 40 minutes per session, lasting at least 12 weeks, with a total weekly dose of 460–620 MET min/week, is recommended to achieve the most pronounced blood pressure-lowering effects.

## Introduction

Hypertension is one of the most prevalent chronic diseases worldwide and a significant risk factor for severe conditions such as cardiovascular disease and stroke, posing a substantial global public health burden (Lim et al., 2012). According to the Global Report on Hypertension released by the World Health Organization (WHO) in 2025, the number of adults aged 30–79 years living with hypertension has reached 1.4 billion globally, and this figure continues to rise (Farrar & Frieden, 2025). In China, as of 2024, approximately 271.5 million adults aged 30–79 years were affected by hypertension, corresponding to a prevalence of about 29% (Farrar & Frieden, 2025). Moreover, cardiovascular and cerebrovascular diseases account for more than 40% of all deaths in China, with approximately 70% of stroke-related deaths and about 50% of myocardial infarctions being closely associated with hypertension (Diseases, Health & China, 2025). Pharmacological therapy remains one of the primary approaches for the treatment and management of hypertension (Ou, Xue & Zhang, 2024). However, long-term medication use may be accompanied by adverse effects and an increased risk of drug dependence (Yin et al., 2023). Therefore, exploring safe and effective non-pharmacological intervention strategies is of great importance. Exercise interventions are widely recommended as non-pharmacological approaches to lower blood pressure and improve lipid profiles, thereby reducing overall cardiovascular risk (Hanssen et al., 2022).

The World Health Organization has described walking as “the best exercise in the world.” Brisk walking is an aerobic exercise modality that lies between leisurely walking and running, characterized by walking at a relatively fast pace, typically 4–6 km/h or approximately 100–120 steps per minute (Hennekens, 2000). Evidence indicates that walking can improve lipid profiles, control blood pressure, and enhance mood, while reducing the risk of various diseases, such as diabetes and cardiovascular diseases (Banach et al., 2023; Cigarroa et al., 2023; Hanson & Jones, 2015). Walking offers several advantages, including moderate intensity, ease of implementation, low cost, broad applicability, and minimal requirements for facilities or equipment. It can be performed in home, community, and laboratory settings, demonstrating high feasibility, scalability, and adherence (Zhaofeng, Lianlin & Lu, 2020). Previous studies have shown that brisk walking significantly improves blood pressure in patients with hypertension (Li, Wei & Can, 2018; Wu et al., 2023). However, the optimal brisk walking dose required to achieve meaningful blood pressure reduction remains unclear (Malem, Ristiani & Ali Puteh, 2024). This uncertainty, to some extent, limits the precise application of brisk walking in hypertensive populations and constrains the development of evidence-based exercise prescriptions and clinical practice guidelines.

Compared with traditional meta-analysis, Bayesian approaches offer greater flexibility through probabilistic inference and incorporation of prior information, potentially improving estimate stability (Gelman et al., 2013). Notably, Bayesian dose–response modelling enables characterization of nonlinear relationships, better handling of heterogeneity, and identification of potential optimal dose ranges beyond categorical comparisons (Röver et al., 2021). Therefore, this study employed a Bayesian multilevel modelling approach to conduct a systematic review and meta-analysis, evaluating the effects of brisk walking on blood pressure in patients with hypertension, elucidating the dose–response relationship, and determining the optimal exercise dose range to achieve the most significant blood pressure-lowering effect.

## Methods

This study was designed and conducted in accordance with the Preferred Reporting Items for Systematic Reviews and Meta-Analyses (PRISMA) guidelines (Shamseer et al., 2015). The review protocol was registered in the PROSPERO database (registration number: CRD42024582852).

### Search strategy

Databases including China National Knowledge Infrastructure (CNKI), VIP Information (VIP), Wanfang Data, the Chinese Biomedical Literature Database (CBM), PubMed, EBSCO (MEDLINE), Embase, the Cochrane Library and Web of Science were systematically searched. The search period covered from database inception to December 2025, with the last search conducted on December 30, 2025. Both controlled vocabulary terms and free-text terms were used in combination. The search terms included brisk walking, vigorous walking, hypertension, high blood pressure, essential hypertension, and primary hypertension. To identify randomized controlled trials (RCTs) examining the effects of brisk walking in patients with hypertension, the reference lists of the included studies were manually screened for additional relevant publications. The whole search strategy for each database is presented in Supplemental Materials 1.

### Literature inclusion, exclusion criteria, and outcome indicator

The inclusion criteria for the literature in this study were formulated according to the PICOS framework. Detailed information is available in Table 1.

Exclusion criteria: ① Studies not published in Chinese or English; ② Duplicate publications; ③ Studies for which relevant data could not be reliably extracted or full texts were unavailable; ④ Non-peer-reviewed publications; ⑤ Animal studies, cross-sectional studies, and other non-clinical or non-interventional studies.

### Data extraction

Literature screening and data extraction were independently conducted by two researchers (JW and LM), who cross-verified all extracted information. Any discrepancies were resolved through discussion or by consultation with a third researcher (YY) when consensus could not be reached. During the screening process, titles were first reviewed to exclude clearly irrelevant studies, followed by a detailed assessment of abstracts and full texts to determine eligibility. Data extraction focused on the following aspects: (1) basic characteristics of the included studies (*e.g.*, country, publication year, and first author); (2) participant characteristics (*e.g.*, sample size and age); (3) detailed descriptions of the intervention protocols; (4) information relevant to risk-of-bias assessment; and (5) outcome measures and corresponding pre- and post-intervention data.

**Table 1:** PICOS framework.

Parameter	Defined criteria for the current study
P (population)	adults ≥18 years of age with hypertension
I (intervention)	Brisk Walking
C (comparison)	Others training
O (outcomes)	BP (blood pressure): Systolic Blood Pressure (SBP), Diastolic Blood Pressure (DBP)
S (study design)	Randomized controlled trials

For studies that reported results only in graphical form, we first attempted to contact the authors to obtain the original data. In cases where no response was received, data were extracted using *WebPlotDigitizer4.1* (https://automeris.io/WebPlotDigitizer) to minimize potential data loss.

### Assessment of risk of bias for study Quality assessment

The methodological quality of the included studies was independently assessed by two reviewers (QL and LM), who evaluated the risk of bias and cross-checked the results. In cases of disagreement, a third reviewer was consulted to resolve the issue. (YY) The risk of bias was independently assessed using the Cochrane Risk of Bias tool for randomized trials, version 2 (RoB 2), which comprises five domains (Sterne et al., 2019). The overall quality of evidence was graded using the Grading of Recommendations, Assessment, Development, and Evaluation (GRADE) system (Salanti et al., 2014). There was excellent agreement between the two reviewers, with an overall concordance rate of 94% and a Cohen’s kappa value of 0.84, indicating almost perfect agreement (Landis & Koch, 1977).

### Data coding and statistical analysis

To evaluate the dose–response relationship between physical activity dose and blood pressure, the total weekly physical activity dose was defined as the metabolic equivalent of task (MET) for the specific activity multiplied by the duration of each session (minutes) and the exercise frequency. The final value was expressed as MET min/week. The MET values for brisk walking were estimated based on the 2024 *Adult Compendium of Physical Activities*, taking into account the reported characteristics of the intervention (*e.g.*, walking speed, intensity description, or exercise prescription) in each study (Herrmann et al., 2024).

Blood pressure improvement was used as the effect indicator, and Hedges’g was selected as the effect size. The calculation formula was as follows:

(1) \begin{eqnarray*}Hedge{s}^{{^{\prime}}}g= \left( \frac{ \left( {M}_{1}-{M}_{2} \right) }{{S}_{P}} \right) \times \left[ 1- \frac{3}{ \left( {N}_{1}-{N}_{2} \right) -9} \right] \end{eqnarray*}

where M_1_ and M_2_ represent the post-intervention mean depression scores of the intervention and control groups, respectively; N_1_ and N_2_ denote the sample sizes of the intervention and control groups, respectively; and S_*p*_ represents the pooled standard deviation, calculated as follows:

(2) \begin{eqnarray*}{S}_{P}=\sqrt{ \frac{({N}_{1}-1){S}_{1}^{2}+({N}_{2}-1){S}_{2}^{2}}{{N}_{1}+{N}_{2}-2} }\end{eqnarray*}

where *S_1_* and *S_2_* represent the standard deviations of the two groups.

A Bayesian meta-analysis was conducted using the brms package in R software (version 4.4.3), with probability statements reported to facilitate intuitive interpretation of the results. A Bayesian hierarchical model was constructed, in which effect sizes were nested within studies, and posterior distributions of the estimated parameters were obtained. Weakly informative priors were specified to improve model stability while limiting undue prior influence. Specifically, normal priors (Normal(0, 1)) were assigned to the intercept and regression coefficients (fixed effects), reflecting no strong prior assumptions regarding the direction or magnitude of the effects. For between-study heterogeneity (*τ*, the standard deviation of the random effects), a half-Cauchy prior with a scale parameter of 1 (Half-Cauchy(0, 1)) was used (Röver et al., 2021). All analytical inferences were based on posterior distributions generated using Hamiltonian Markov chain Monte Carlo (Hamiltonian MCMC) methods (Etzioni & Kadane, 1995), and uncertainty in the estimates was expressed as 95% credible intervals (CrI). Given the variability in physical activity interventions and study populations, we assumed within- and between-study heterogeneity in intervention effect estimates; therefore, a random-effects model was adopted. Model convergence and validity were assessed using the potential scale reduction factor (PSRF), with values <1.01 indicating adequate convergence (Brooks & Gelman, 1998). The accurate study-specific effect sizes were extracted using the ranef function, and their deviations from the pooled effect were estimated. Effect sizes were interpreted based on the absolute value of Hedges’g, with values <0.2 indicating minor effects, 0.2−0.8 indicating moderate effects, and ≥0.8 indicating large effects (Hedges & Olkin, 1985). In addition to standardized effect sizes (Hedges’ g), absolute effects were quantified using mean differences (MD) in SBP and DBP to enhance clinical interpretability.

To assess publication bias and perform regression analyses, funnel plots and Egger’s test were conducted using the metafor package in R (Lin et al., 2018). Meta-regression analyses were performed to explore the potential moderating effects of covariates, including age, sex, intervention duration, frequency, intensity, and intervention period. In addition, the dosresmeta package was used to construct nonlinear dose–response relationship models. Data visualization was carried out using the ggplot2 package. Furthermore, the fail-safe N method was applied to evaluate the risk of publication bias. When N_fs_ ≥ 5k + 10, the presence of potential publication bias is suggested. The calculation formula is as follows:

(3) \begin{eqnarray*}{N}_{\mathrm{fs}}= \frac{{ \left( \Sigma {\mathrm{Z}}_{\mathrm{i}} \right) }^{2}}{{\mathrm{Z}}_{\mathrm{a}}} -K.\end{eqnarray*}

If publication bias was detected (Egger’s test *P* ≤ 0.05), the trim-and-fill method was applied to adjust the effect size. If the direction of the effect estimate did not change after adjustment, publication bias was considered to have a minimal impact on the results (Duval & Tweedie, 2000).

## Results

### Study search results

**Figure 1:**
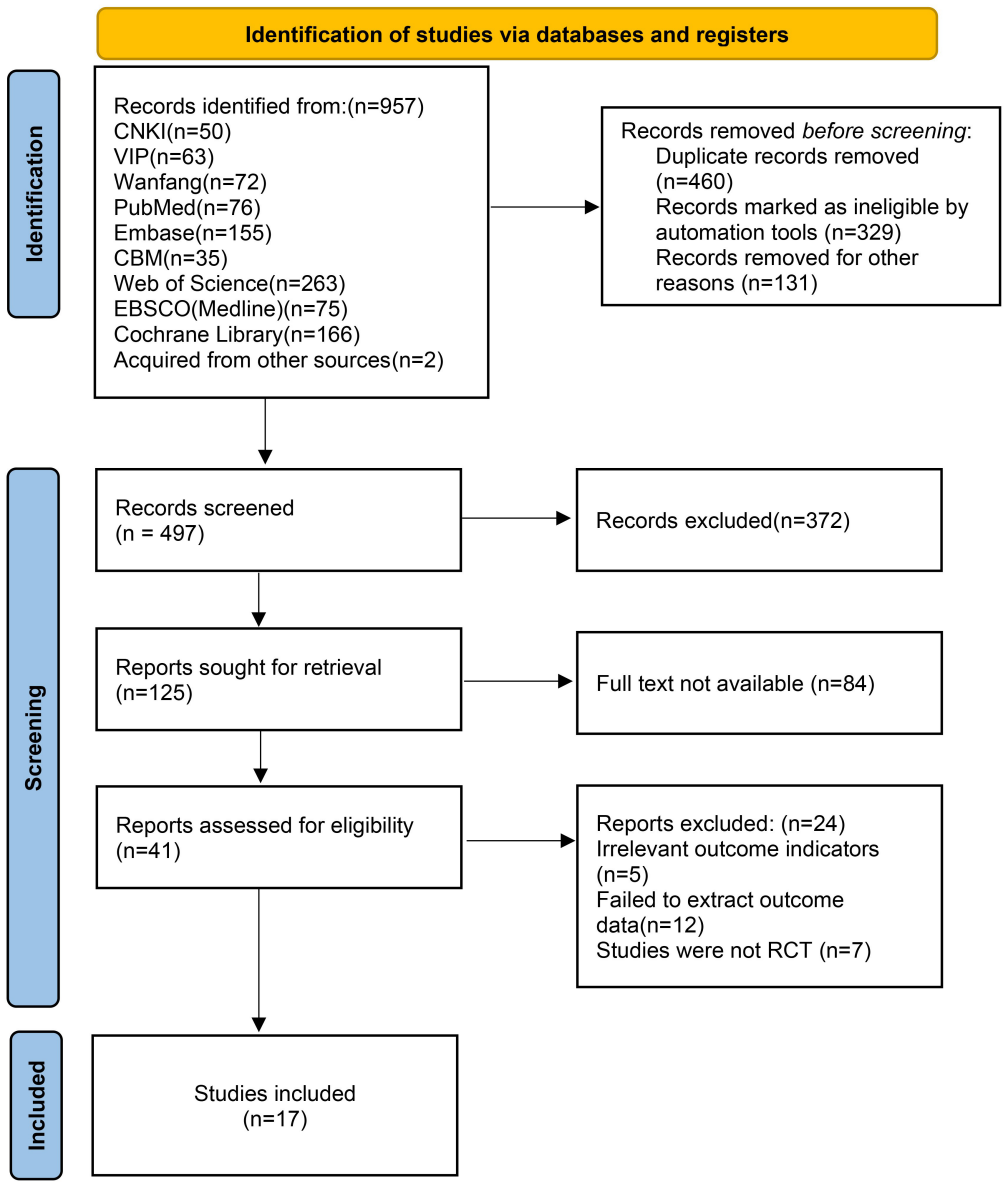
Literature review flowchart.

Through database searching, a total of 955 articles were initially identified, with an additional two articles obtained from other sources. All retrieved records were imported into EndNote X9 for management, and duplicate records were removed, leaving 497 articles. After screening titles and abstracts, 125 articles were retained for full-text review. Following full-text assessment, 41 articles were considered potentially eligible. Of these, 24 articles were excluded for failing to meet the inclusion and exclusion criteria, leaving 17 articles for the final analysis. The literature screening process and results are presented in Fig. 1.

### Study characteristics

A total of 17 articles (Liu & Zhuo, 2021; En-hong et al., 2017; Xu, 2017; Li et al., 2018; Li, Wei & Can, 2018; Humphrey & Ogu, 2021; Chen & Mao, 2016; Ko et al., 2021; Xia et al., 2013; Xia et al., 2011; Zhang et al., 2012; Wang & Wang, 2024; Rachmawati, Sugiarto & Hastuti, 2019; Huang, 2020; Wu et al., 2023; Yun, 2015; Wu & Lin, 2021) were included, comprising an overall sample size of 1,493 participants, with 758 participants in the intervention group and 735 in the control group. Most of the included studies were conducted in China, with relatively few studies originating from other regions (*e.g.*, Canada, Indonesia, and Nigeria). The duration of the interventions ranged from 2 to 24 weeks, with intervention frequencies of three to seven sessions per week (Table 2). Regarding bias in outcome measurement, 76.48% of the studies were rated as having a “low risk” of bias, 17.64% were judged as having “some concerns,” and 5.88% were assessed as having a “high risk” of bias (Fig. S1).

**Table 2:** Description of studies included in the meta-analysis.

**Study**	**Country**	**Age (years)**	**Sample size (T/C, n)**	**Intervention (T/C)**	**Time (min)**	**Frequency (times/week)**	**Duration** ** (weeks)**	**Outcome**
Li et al., 2018	China	58 ± 2 57 ± 2	23/23	Brisk Walking/No intervention	45–60	3	12	SBP/DBP
En-Hong et al., 2017	China	59.20 ± 3.45 57.18 ± 1.30	110/120	Brisk Walking/No intervention	–	7	8/12	SBP/DBP
Liu & Zhuo, 2021	China	55.8 ± 5.1 56.1 ± 4.6	23/23	Brisk Walking/No intervention	30–30	3–5	12/24	SBP/DBP
Wang & Wang, 2024	China	40∼69	33/22	Brisk Walking/No intervention	30–60	3	4/8/12	SBP/DBP
Ko et al., 2021	Canada	61.2 ± 15 61.9 ± 8.4	20/20	Brisk Walking/Stretching	30	5	8	SBP/DBP
Li, Wei & Can, 2018	China	58 ± 2 57 ± 2	23/23	Brisk Walking/No intervention	45–60	3	12	SBP/DBP
Wu et al., 2023	China	57.34 ± 7.80 58.33 ± 7.84	77/66	Brisk Walking/No intervention	45–60	3	12	SBP/DBP
Xia et al., 2011	China	68.07 ± 7.03 68.07 ± 7.03	44/44	Brisk Walking/No intervention	30	5	24	SBP/DBP
Zhang et al., 2012	China	68.07 ± 7.03 68.07 ± 7.03	44/44	Brisk Walking/No intervention	30	5	24	SBP/DBP
Xu, 2017	China	57.19 ± 7.16 56.31 ± 6.98	20/20	Brisk Walking/No intervention	20–40	7	24	SBP/DBP
Xia et al., 2013	China	68.07 ± 7.03 68.07 ± 7.03	44/44	Brisk Walking/No intervention	30	5	24	SBP/DBP
Rachmawati, Sugiarto & Hastuti, 2019	Indonesia	40–60	15/15	Brisk Walking/No intervention	30	4	2	SBP/DBP
Wu & Lin, 2021	China	46.2 ± 6.6 46.3 ± 5.1	50/50	Brisk Walking/No intervention	30–60	3–5	12	SBP/DBP
Humphrey & Ogu, 2021	Nigeria	36–45	20/13	Brisk Walking/No intervention	30	5	12	SBP/DBP
Chen & Mao, 2016	China	65.16 ± 7.43 65.36 ± 7. 51	134/134	Brisk Walking/No intervention	–	5	24	SBP/DBP
Huang, 2020	China	66.08 ± 1.43 66.01 ± 1.38	50/46	Brisk Walking/No intervention	–	5	12	SBP/DBP
Yun, 2015	China	46.3 ± 6.50 45.8 ± 5.71	28/28	Brisk Walking/No intervention	25–45	7	12	SBP/DBP

### Effects of brisk walking

**Figure 2:**
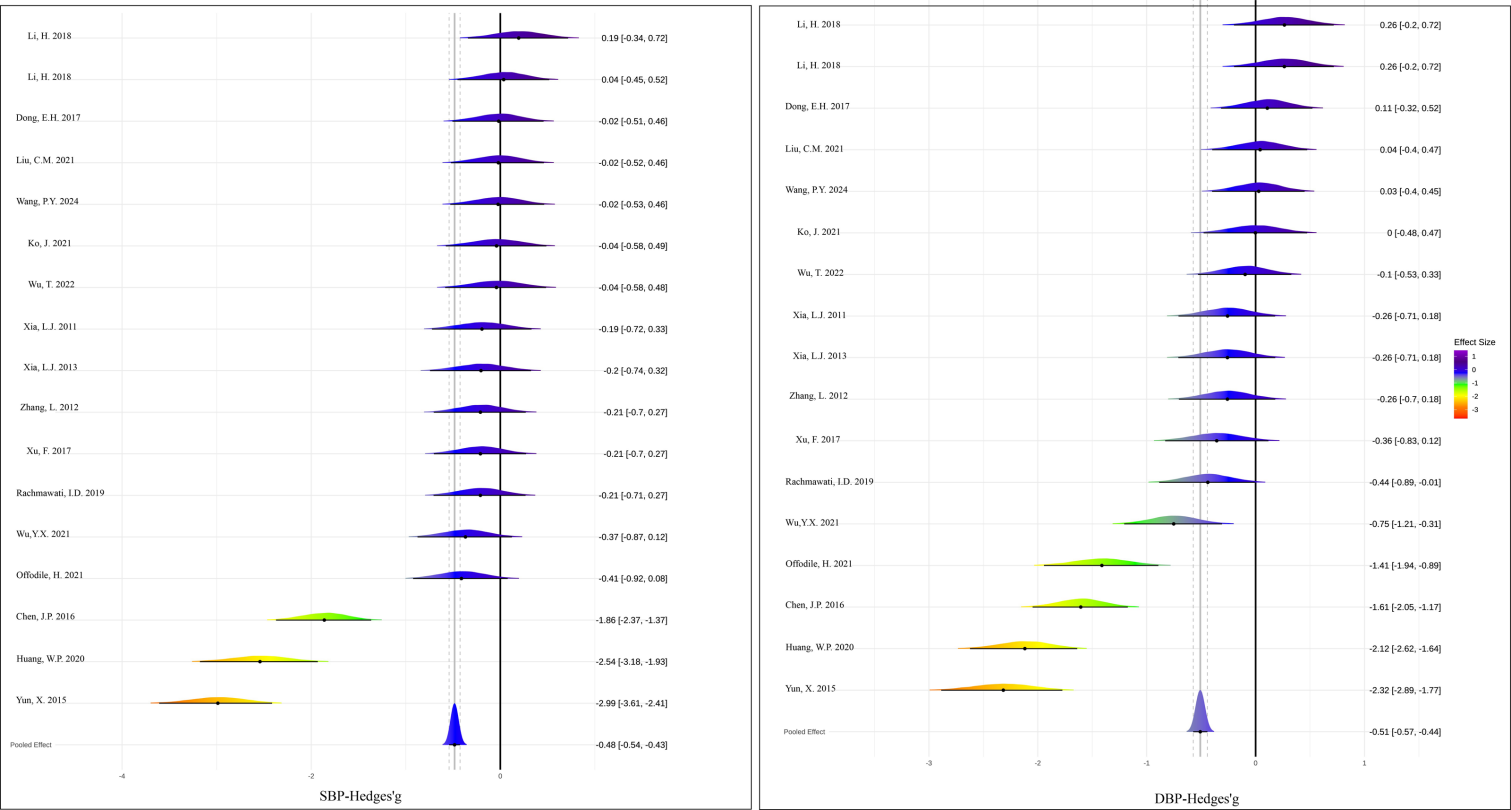
Forest plots of the effects of brisk walking on SBP and DBP. Hedges’g measures the effect size.

Brisk walking significantly reduced SBP (Hedges’ g = −0.48, 95% credible interval (CrI) [−0.54 to −0.43]), with substantial between-study heterogeneity (*τ* = 1.00, 95% CrI [0.70–1.45]), and good model convergence (RSRF = 1.00). Brisk walking also significantly reduced DBP (Hedges’ g = −0.51, 95% CrI [−0.58 to −0.44]), with moderate between-study heterogeneity (*τ* = 0.87, 95% CrI [0.61–1.28]), and satisfactory model convergence (RSRF = 1.00) (Fig. 2; Table 3).

**Table 3:** Bayesian meta-analysis of the effects of brisk walking on SBP and DBP.

**Variable**		**No.of** **Trials/total**		**Sample size**		**SE**		**Hedges’ g (95% CrI)**		**RSRF**		**SD**_**Intercept**_ ** (95%CrI)**		
	SBP		17		1,999		0.19		−0.48 [−0.54, −0.43]		1.00		1.00 [0.70, 1.45]		
	DBP		17		1,999		0.17		−0.51 [−0.57, −0.44]		1.00		0.87 [0.61, 1.28]		
SBP	**Single-session time**						
	**≤40 min**	12/17		897		0.19		**−0.61 [−0.69, −0.52]**		1.00		0.75 [0.48, 1.21]	
	>40 min	4/17		243		0.76		−0.52 [−0.70, −0.35]		1.00		1.71 [0.83, 3.68]	
	**Frequency**						
	**≤4** ** times/week**		7/17		552		0.10		**−0.63 [−0.73, −0.53]**		1.00		0.23 [0.11, 0.49]	
	>4 times/week		10/17		1,111		0.32		−0.41 [−0.48, −0.34]		1.00		1.26 [0.80, 2.06]	
	**Duration**						
	≤12 weeks	12/17		1,091		0.27		−0.44 [−0.51, −0.37]		1.00		1.13 [0.74, 1.78]	
	**>12** ** weeks**	6/17		618		0.31		**−0.59 [−0.70, −0.48]**		1.00		0.81 [0.43, 1.60]	
DBP	**Single-session time**						
	**≤40 min**	12/17		897		0.19		**−0.55 [−0.65, −0.45]**		1.00		0.77 [0.50, 1.22]	
	>40 min	4/17		243		0.61		−0.14[−0.31, 0.03]		1.00		1.32 [0.62, 2.92]	
	**Frequency**						
	≤4 times/week	7/17		552		0.16		−0.36[−0.47, −0.26]		1.00		0.44 [0.23, 0.85]	
	**>4 times/week**	10/17		1,111		0.27		**−0.60[−0.69, −0.52]**		1.00		1.02 [0.63, 1.67]	
	**Duration**						
	≤12 weeks	12/17		1,091		0.23		−0.44 [−0.51, −0.36]		1.00		0.99 [0.65, 1.55]	
	**>12** ** weeks**	6/17		618		0.27		**−0.80 [−0.94, −0.65]**		1.00		0.70 [0.37, 1.39]	

Brisk walking interventions significantly reduced SBP, with a pooled mean difference of −6.59 mmHg (95% CI [−8.81 to −4.37]). Similarly, brisk walking was associated with a significant reduction in DBP, with a pooled mean difference of −4.67 mmHg (95% CI [−6.26 to −3.08]) (Figs. S2–S3).

**Figure 3:**
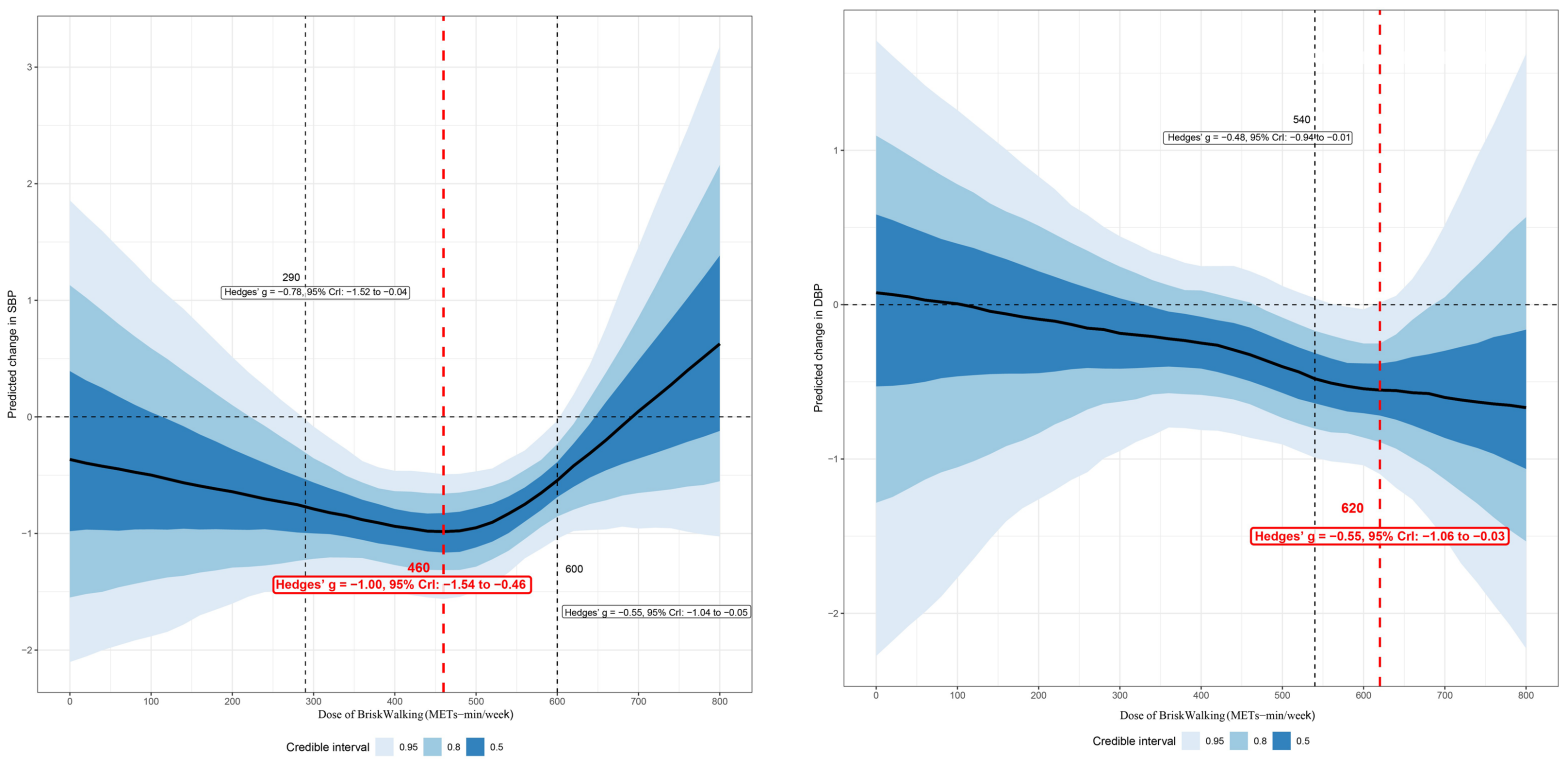
Dose–response relationship between brisk walking and SBP and DBP. Hedges’g measures the effect size.

A “U” shaped dose–response relationship was observed between brisk walking and SBP. The minimum effective dose required for a significant reduction was 290 MET min/week (Hedges’ g = −0.78, 95% credible interval (CrI) [−1.52 to −0.04]). The optimal effect was observed at 460 MET min/week (Hedges’ g = −1.00, 95% CrI [−1.54 to −0.46]). The maximum tolerable dose was approximately 600 MET min/week (Hedges’ g = −0.55, 95% CrI [−1.04 to −0.05]). In contrast, a non-linear inverse dose–response relationship was observed between brisk walking and DBP, with DBP decreasing as walking dose increased, followed by a gradual attenuation of the effect at higher doses. The minimum dose associated with a significant reduction was approximately 540 MET min/week (Hedges’ g = −0.48, 95% CrI [−0.94 to −0.01]), while the optimal dose was approximately 620 MET min/week, at which the blood pressure-lowering effect reached its maximum (Hedges’ g = −1.55, 95% CrI [−1.06 to −0.03]) (Fig. 3).

### Subgroup analysis

To further explore the effects of brisk walking on SBP and DBP, subgroup analyses were conducted based on single-session duration, intervention frequency, and intervention period.SBP:① Single-session duration: ≤40 min (Hedges’ g = −0.61, 95% credible interval (CrI) [−0.69 to −0.52]) and >40 min (Hedges’ g = −0.52, 95% CrI [−0.70 to −0.35]); ② Exercise frequency: ≤4 sessions/week (Hedges’ g = −0.63, 95% CrI [−0.73 to −0.53]) and >4 sessions/week (Hedges’ g = −0.41, 95% CrI [−0.48 to −0.34]); ③ Intervention period: ≤12 weeks (Hedges’ g = −0.44, 95% CrI [−0.51 to −0.37]) and >12 weeks (Hedges’ g = −0.59, 95% CrI [−0.70 to −0.48]). DBP: ① Single-session duration: ≤40 min (Hedges’ g = −0.55, 95% CrI [−0.65 to −0.45]) and >40 min (Hedges’ g = −0.14, 95% CrI [−0.31 to 0.03]); ② Exercise frequency: ≤4 sessions/week (Hedges’ g = −0.36, 95% CrI [−0.47 to −0.26]) and >4 sessions/week (Hedges’ g = −0.60, 95% CrI [−0.69 to −0.52]); ③ Intervention period: ≤12 weeks (Hedges’ g = −0.44, 95% CrI [−0.51 to −0.36]) and >12 weeks (Hedges’ g = −0.80, 95% CrI [−0.94 to −0.65]) (Table 3).

### Publication bias

Bias analyses for SBP and DBP indicated some asymmetry in the funnel plots. Egger’s test yielded t = −3.0011 (*P* = 0.0042) for SBP and t = −2.8026 (*P* = 0.0072) for DBP, suggesting potential small-study effects or publication bias. The trim-and-fill method was subsequently applied to adjust for potential publication bias; however, no missing studies were identified, and the pooled effect estimates remained unchanged after adjustment. Although this suggests that the influence of publication bias on the overall effect size may be limited, it cannot fully exclude the presence of bias, particularly given the known limitations of the trim-and-fill method in detecting funnel plot asymmetry. The Nfs values for SBP and DBP were 12,829.39 and 11,721.53, respectively, both substantially exceeding the decision threshold (5k + 10 = 270). Nevertheless, the presence of potential publication bias may lead to an overestimation of the true effect size. Therefore, despite the apparent stability indicated by the Nfs results, the statistically significant Egger’s test suggests that the findings should be interpreted with caution.

### Meta-regression analysis

To explore potential sources of heterogeneity in effect sizes, meta-regression analyses were conducted, incorporating covariates including age, sex, single-session exercise duration, exercise frequency, exercise intensity, and intervention period, to examine their moderating effects on intervention outcomes. The results showed that exercise frequency was the only significant moderator of the intervention effect on systolic blood pressure (SBP) (*β* = −0.384, 95% CI [−0.710 to −0.060], *P* = 0.020). For diastolic blood pressure (DBP), both exercise frequency (*β* = −0.495, 95% CI [−0.730 to −0.260], *P* < 0.001) and exercise intensity (*β* = −0.004, 95% CI [−0.010 to −0.000], *P* = 0.023) were identified as significant moderators of the intervention effect, whereas the moderating effects of the other covariates did not reach statistical significance (Figs. S4–S5).

### Level of evidence

According to the GRADE assessment results presented in the table, the certainty of evidence for both systolic blood pressure (SBP) and diastolic blood pressure (DBP) was rated as low. Therefore, the overall quality of evidence is limited, and the conclusions should be interpreted with caution (see Table 4).

**Table 4:** GRADE evidence for the effects of brisk walking.

Variable	Risk of Bias	Inconsistency	Indirectness of evidence	Imprecision	Publication bias	Quality of evidence	Hedges’ g (95% CrI)
SBP	No	Serious	No	Serious	No	Low (⊕⊕○○)	−0.48[−0.54, −0.43]
DBP	No	Serious	No	Serious	No	Low (⊕⊕○○)	−0.51[−0.57, −0.44]

**Notes.**

Risk of bias: No: Most information is from results with a low risk of bias. Serious: A crucial limitation for one criterion, or some limitations for multiple estimates of effect. Very Serious: A significant restriction for one or more criteria, sufficient to substantially lower confidence in the estimate of effect.

Inconsistency: Serious: I^2^ > 40%. Very Serious: I^2^ > 80%.

Indirectness of Evidence: No indirectness in the evidence was found in any study.

Imprecision (based on sample size): Serious: *n* < 250 subjects, Very Serious: *n* < 250, and the estimated effect is small or absent.

Publication bias (based on funnel plots): No publication bias was found. Funnel plots are not shown due to the small number of trials.

## Discussion

This study systematically evaluated the effects of brisk walking on blood pressure using a Bayesian dose–response meta-analytic framework. The findings indicate that brisk walking significantly reduced both systolic and diastolic blood pressure, with effect sizes of moderate magnitude. A “U” shaped dose–response relationship was observed between brisk walking and systolic blood pressure, whereas a monotonic negative dose–response relationship was identified for diastolic blood pressure. The most significant improvement in systolic blood pressure occurred at approximately 460 MET min/week, while the maximal reduction in diastolic blood pressure was observed at approximately 620 MET min/week.

Long-term hypertension places the heart and blood vessels under sustained hemodynamic overload, leading to a progressive loss of vascular elasticity and increased arterial stiffness, thereby elevating the risk of cardiovascular and cerebrovascular diseases such as coronary heart disease, myocardial infarction, and stroke (Fuchs & Whelton, 2020). Evidence indicates that a reduction of five mmHg in SBP is associated with a 13% decrease in stroke risk (Blood Pressure Lowering Treatment Trialists’ Collaboration, 2021). Therefore, blood pressure reduction is critical for preventing cardiovascular and cerebrovascular diseases (Valenzuela et al., 2021). Meta-analytic findings demonstrate that, compared with other intervention strategies, brisk walking significantly reduces SBP (Hedges’ g = −0.48, moderate effect; −6.59 mmHg) and DBP (Hedges’ g = −0.51, moderate effect; −4.67 mmHg), which is consistent with previous studies (Malem, Ristiani & Ali Puteh, 2024). Exercise has been shown to enhance parasympathetic nervous system activity while attenuating sympathetic nervous system activity (Higashi & Yoshizumi, 2004), thereby contributing to reduced vascular tone, decreased peripheral vascular resistance, and alleviated cardiac workload. These physiological adaptations help prevent arterial stiffening and ultimately reduce both systolic and diastolic blood pressure (Son et al., 2017). Brisk walking, as a moderate-intensity aerobic exercise, lowers blood pressure by enhancing endothelial function, increasing vascular elasticity, and reducing vascular resistance (Tanaka, 2019). During brisk walking, increased blood flow stimulates endothelial cells to release more nitric oxide (NO) (Muskat et al., 2023). NO is recognized as one of the most essential endothelium-dependent vasodilators and is synthesized from L-arginine (L-Arg) by nitric oxide synthase (NOS) (Tanaka, 2019). In addition, brisk walking indirectly contributes to blood pressure reduction by attenuating sympathetic nervous system activity, improving insulin sensitivity, and alleviating inflammatory responses (Brook et al., 2013). The results of bias assessments further strengthen the credibility of the evidence presented in this study. Although substantial heterogeneity was observed, additional analyses, including funnel plots, Egger’s test, the trim-and-fill method, and the Nfs, were conducted to examine further the robustness of the findings and the risk of publication bias. The results indicated that, despite some indications of publication bias, the overall conclusions of this study remain robust and reliable.

The present study clearly delineated the dose–response relationships between brisk walking and both SBP and DBP. A U-shaped dose–response association was observed between brisk walking and SBP, with a significant antihypertensive effect first detected at approximately 290 MET min/week and reaching its maximum at around 460 MET min/week. When the weekly training volume exceeded 600 MET min/week, the magnitude of blood pressure reduction declined, suggesting that a moderate dose yields the optimal intervention effect. In contrast, both insufficient and excessive exercise doses may fail to provide additional benefits: lower doses may not reach the physiological stimulus threshold, whereas higher doses may attenuate the antihypertensive effect due to exercise-induced fatigue, muscle damage, or increased cardiovascular load (Eijsvogels, Thompson & Franklin, 2018). In contrast, a linear inverse dose–response relationship was observed between brisk walking and DBP. Significant improvements were evident once the training volume exceeded approximately 540 MET min/week, with the most considerable reduction observed at approximately 620 MET min/week. Previous studies have shown that aerobic training reduces diastolic blood pressure as the exercise dose increases, with maximal benefits observed at moderate doses. This effect may be attributable to the cumulative physiological adaptations induced by exercise, including reductions in peripheral vascular resistance, improvements in endothelial function, and enhanced cardiovascular regulatory mechanisms (De Barcelos et al., 2022; Jabbarzadeh Ganjeh et al., 2024). Subgroup analyses further underscore the importance of exercise program design. For SBP, interventions with a session duration of ≤40 min, a frequency of ≤4 sessions per week, and an intervention period of >12 weeks were associated with more pronounced reductions, suggesting that moderate frequency, appropriate session duration, and longer intervention periods are more conducive to achieving stable improvements in systolic blood pressure. In contrast, for DBP, interventions lasting >12 weeks, a frequency of >4 sessions per week, and with session durations of ≤40 min were associated with greater reductions. These findings suggest differential response patterns of SBP and DBP to exercise prescription characteristics, particularly with respect to training frequency and intervention duration. Collectively, the dose–response relationships and subgroup analyses suggest that a brisk walking program consisting of approximately four sessions per week, a session duration not exceeding 40 min, an intervention period longer than 12 weeks, and a total weekly dose of 460-620 MET min/week is the most effective for improving both systolic and diastolic blood pressure.

The results of the meta-regression analysis indicated that exercise frequency was the only significant moderator influencing both SBP and DBP. In contrast, exercise intensity exerted a significant effect only on DBP. Higher exercise frequency was associated with greater reductions in blood pressure, an effect that was particularly pronounced in the DBP models. From a physiological perspective, high-frequency low- to moderate-intensity exercise may effectively reduce diastolic blood pressure by improving vascular compliance, decreasing peripheral vascular resistance, and enhancing autonomic nervous system balance. In addition, the influence of exercise intensity on DBP appears to be mediated by repeated moderate-load stimuli, which may improve microvascular function and endothelial regulatory capacity (Yang et al., 2024; Zhou et al., 2022). Several limitations of this study should be acknowledged. First, most included studies lacked post-intervention follow-up assessments, precluding evaluation of the long-term sustainability of the blood pressure–lowering effects of brisk walking. Second, substantial heterogeneity was observed across studies, and some trials exhibited potential methodological shortcomings during study design and implementation, including measurement bias, performance bias, and reporting bias (*e.g.*, unclear allocation concealment or blinding procedures). These issues may have reduced the overall quality of evidence and affected the stability of the findings. Furthermore, many studies did not adequately report potential confounding factors, such as antihypertensive medication use, smoking and alcohol consumption, baseline physical activity levels, and nutritional status, thereby limiting further exploration of their moderating or confounding effects. In addition, the inclusion of studies published only in English and Chinese may have introduced potential language bias and limited the comprehensiveness of the evidence.

Therefore, although the present study demonstrates a significant beneficial impact of brisk walking on blood pressure reduction, its conclusions should be further validated through high-quality, rigorously designed clinical trials with long-term follow-up. Future research should consider expanding the range of included languages and incorporating multilingual translation strategies to minimize potential bias and enhance the robustness of the findings.

In conclusion, the present study suggests that brisk walking is associated with beneficial effects on blood pressure. Based on dose–response modelling, a potential optimal dose range of approximately 460–620 MET min/week was identified, within which the greatest reductions in both systolic and diastolic blood pressure were observed. In practical terms, this corresponds to a moderate-intensity brisk walking program performed approximately four times per week, with each session lasting ≤40 min and sustained for more than 12 weeks, resulting in a total weekly exercise volume of approximately 150–210 min. However, given the low certainty of evidence and the heterogeneity across studies, these findings should be interpreted with caution, and the proposed dose range should be considered an approximate reference rather than a definitive prescription. For individuals with lower physical fitness levels, initiating training at a lower dose and gradually increasing the volume toward this range may represent a feasible approach to ensure safety and promote adherence (Table 5).

**Table 5:** Recommended weekly exercise dose for brisk walking.

Level/Phase	Primary goal	Frequency (days/week)	Session duration (mins/session)	Total weekly time (mins/week)	Approx. Weekly Dose (MET-min/week)^a^
Level 1 Foundational Phase	To build exercise habits, ensure safety, and achieve the minimum effective dose for blood pressure reduction	3–4	20–30	60–120	∼290–460
Level 2 Optimal Therapeutic Phase	To achieve the maximum improvement in both systolic and diastolic blood pressure	4–5	30–40	150–200	∼460–620
Level 3 Maintenance Phase	To sustain long-term blood pressure reduction and improve cardiovascular endurance	5–6	40–50	200–300+	≥600

**Notes.**

a Dose calculation notes: The MET dose was calculated based on moderate-intensity brisk walking (approximately 3–5 METs) using the following formula: MET min/week = MET × minutes × sessions. A total weekly dose of 460–620 MET min/week represents the combined optimal range for improving both systolic and diastolic blood pressure. Specifically, the maximal improvement point for SBP was observed at 460 MET min/week, whereas the maximal improvement point for DBP occurred at 620 MET min/week. A dose of 290 MET min/week was identified as the minimum effective dose for reducing SBP, while doses ≥600 MET min/week represent the upper tolerable high-dose range.

### Clinical messages

(1) This study provides robust scientific evidence for the development of clear and practical brisk walking exercise prescriptions for older adults.

(2) The findings suggest that routine, low-cost brisk walking may serve as a widely applicable non-pharmacological intervention for blood pressure management.

(3) The proposed exercise prescription offers a scalable and practical framework for community-based interventions, hypertension management programs, and personalized exercise guidance.

##  Supplemental Information

10.7717/peerj.21478/supp-1 Supplemental Information 1 Supplemental tables and figures

10.7717/peerj.21478/supp-2 Supplemental Information 2 PRISMA checklist

10.7717/peerj.21478/supp-3 Supplemental Information 3 PRISMA abstract checklist

## Additional Information and Declarations

### Data Availability

The following information was supplied regarding data availability: This is a Systematic review/Meta-analysis.
