# Supplementary material for: The optimal dose of brisk walking for improving blood pressure in hypertensive patients: a systematic review and bayesian meta-analysis of randomized controlled trials
Source: PeerJ. 2026 Jun 30;14:e21478. doi: 10.7717/peerj.21478 (PMC13330745; doi:10.7717/peerj.21478)
Supplement: Supplemental Information 1 [file peerj-14-21478-s001.docx]

**Supplementary materials**

**Search strategy in PubMed on January 10, 2026**

| 1 | ((brisk walking) OR (vigorous walking)) AND (((((hypertension[MeSH Terms]) OR (high blood pressure[Title/Abstract])) OR (essential hypertension[Title/Abstract])) OR (primary hypertension[Title/Abstract]))) | 76 |
| --- | --- | --- |

**Search strategy in Embase on January 10, 2026**

| 1 | 'brisk walking' OR 'vigorous walking' | 1302 |
| --- | --- | --- |
| 2 | 'hypertension'/exp | 1225414 |
| 3 | 'hypertension':ab,ti OR 'high blood pressure':ab,ti OR 'essential hypertension':ab,ti OR 'primary hypertension':ab,ti | 863187 |
| 4 | #2 OR #3 | 1471237 |
| 5 | #1 AND #4 | 155 |

**Search strategy in Web of Science on January 10, 2026**

| 1 | (ALL=(brisk walking)) OR ALL=(vigorous walking) | 3920 |
| --- | --- | --- |
| 2 | (((TS=((hypertension)) OR TS=(high blood pressure)) OR TS=(essential hypertension)) OR TS=(primary hypertension)) | 709807 |
| 3 | #1 AND #2 | 263 |

**Search strategy in Cochrane Library on January 10, 2026**

| #1 | MeSH descriptor: [Hypertension] explode all trees | 25727 |
| --- | --- | --- |
| #2 | (high blood pressure):ti,ab,kw OR (essential hypertension):ti,ab,kw OR (primary hypertension):ti,ab,kw | 62528 |
| #3 | (brisk walking) OR (vigorous walking) | 1729 |
| #4 | #1 or #2 | 76058 |
| #12 | #3 and #4 | 166 |

**Search strategy in EBSCO_(Medline)_ on January 10, 2026**

| S1 | TX brisk walking OR TX vigorous walking | 1636 |
| --- | --- | --- |
| S2 | SU hypertension OR XB high blood pressure OR XB essential hypertension OR XB primary hypertension | 421664 |
| S3 | S1 AND S2 | 75 |

**Search strategy in CNKI on January 10, 2026**

| 1 | TKA=( brisk walking + vigorous walking)*( hypertension + essential hypertension) | 49 |
| --- | --- | --- |

**Search strategy in VIP on January 10, 2026**

| 1 | U=( brisk walking + vigorous walking)*( hypertension + essential hypertension) | 63 |
| --- | --- | --- |

**Search strategy in wangfang on January 10, 2026**

| 1 | ALL=( brisk walking or vigorous walking)and(hypertension or essential hypertension) | 72 |
| --- | --- | --- |

**Search strategy in CBM on January 10, 2026**

| 1 | (("brisk walking" [all fields: intelligent] OR "vigorous walking" [all fields: intelligent])) AND (("hypertension" [all fields: intelligent] OR "essential hypertension" [all fields: intelligent])) | 35 |
| --- | --- | --- |

**Assessment of risk of bias for study Quality**

**
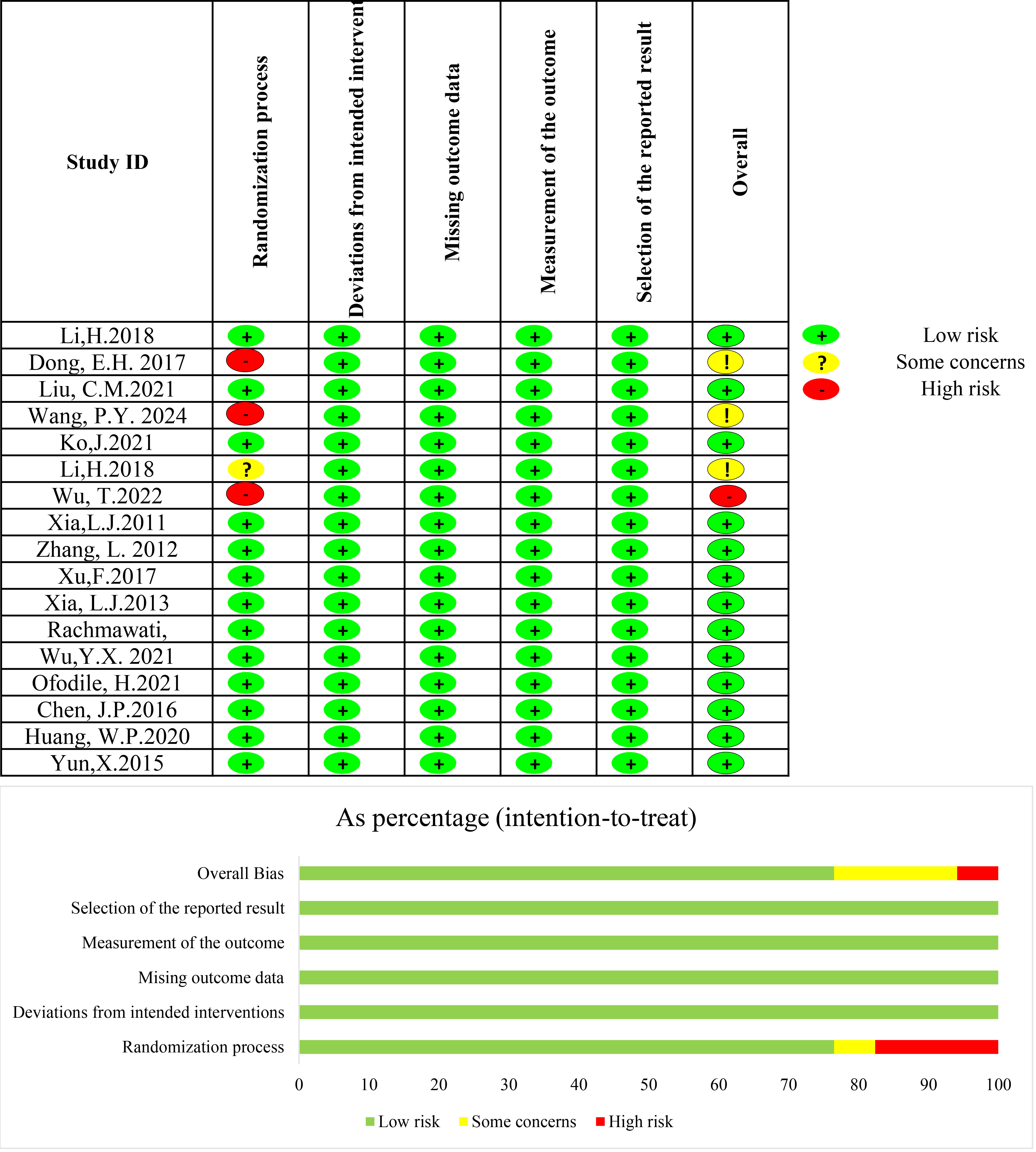
**

**SBP-forest_plot**

**
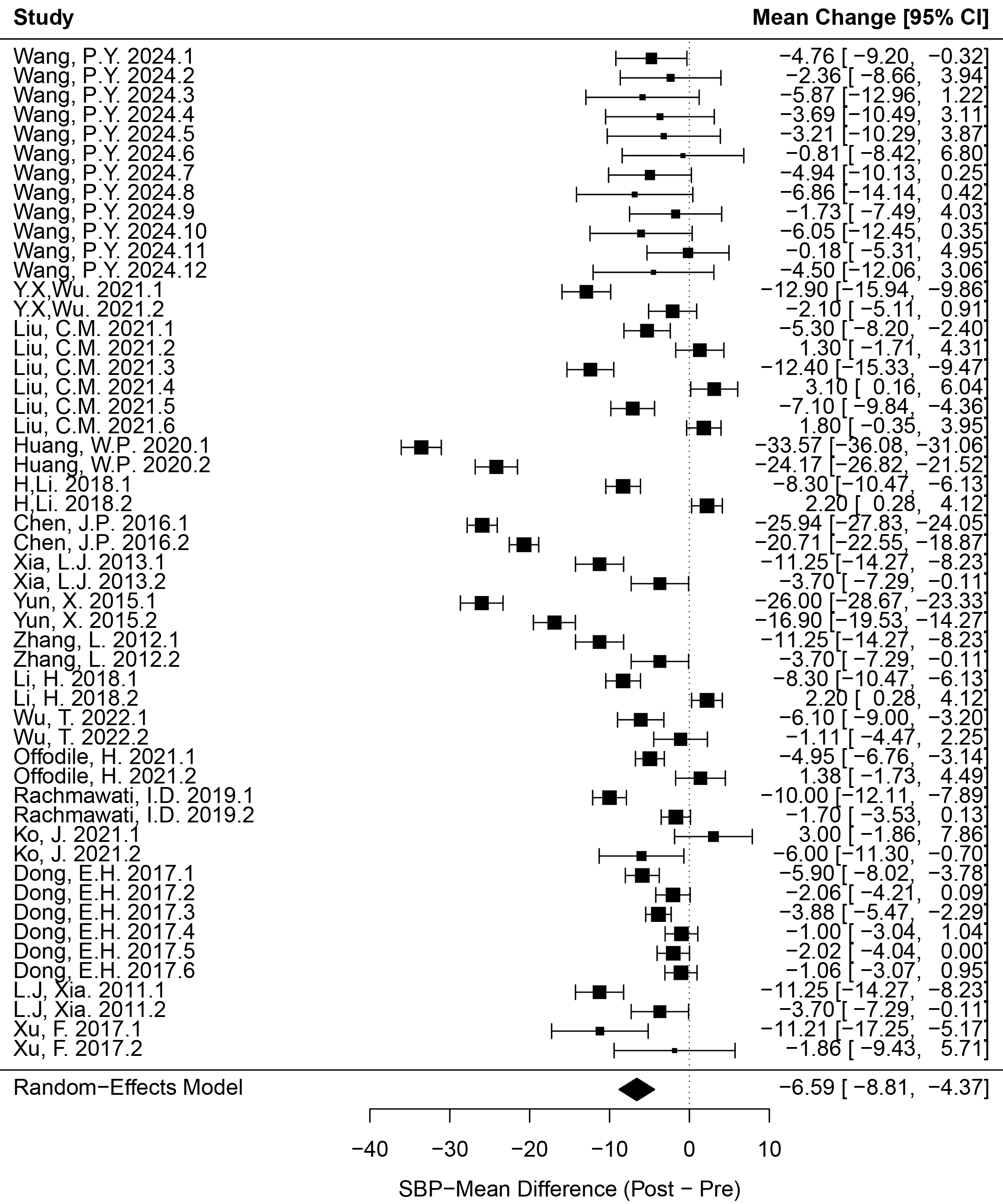
**

**DBP-forest_plot**

**
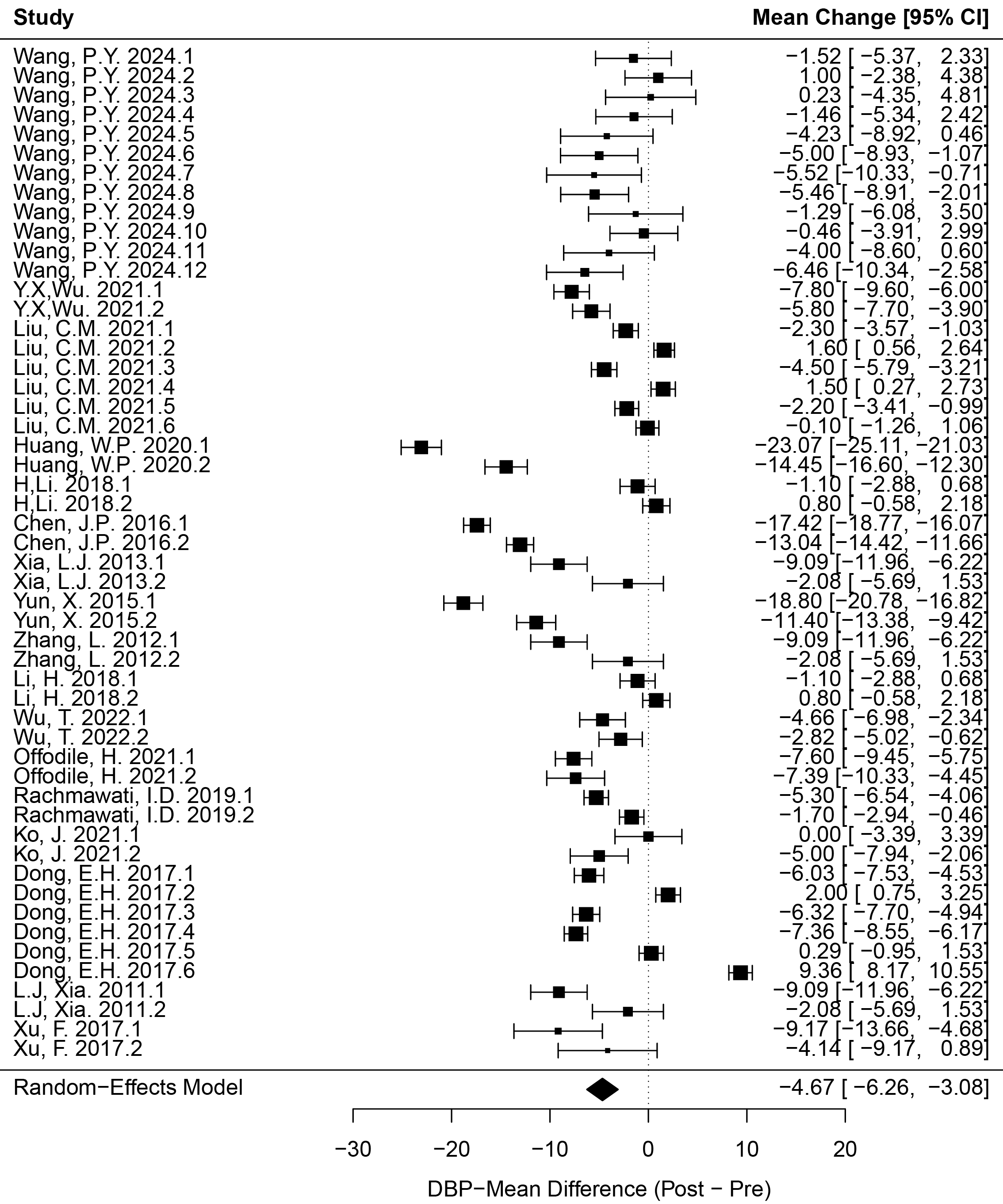
**

**Meta-regression Analysis-SBP**

**

**

**Meta-regression Analysis-DBP**

**

**
